# Influence of Contact Definitions in Assessment of the Relative Importance of Social Settings in Disease Transmission Risk

**DOI:** 10.1371/journal.pone.0030893

**Published:** 2012-02-16

**Authors:** Kirsty J. Bolton, James M. McCaw, Kristian Forbes, Paula Nathan, Garry Robins, Philippa Pattison, Terry Nolan, Jodie McVernon

**Affiliations:** 1 Vaccine and Immunisation Research Group, Melbourne School of Population Health, University of Melbourne, Parkville, Victoria, Australia; 2 School of Psychological Sciences, University of Melbourne, Parkville, Victoria, Australia; 3 Murdoch Childrens Research Institute, Parkville, Victoria, Australia; Cochrane Acute Respiratory Infections Group, Italy

## Abstract

**Background:**

Realistic models of disease transmission incorporating complex population heterogeneities require input from quantitative population mixing studies. We use contact diaries to assess the relative importance of social settings in respiratory pathogen spread using three measures of person contact hours (PCH) as proxies for transmission risk with an aim to inform bipartite network models of respiratory pathogen transmission.

**Methods and Findings:**

Our survey examines the contact behaviour for a convenience sample of 65 adults, with each encounter classified as occurring in a work, retail, home, social, travel or “other” setting. The diary design allows for extraction of PCH-interaction (cumulative time in face-face conversational or touch interaction with contacts) – analogous to the contact measure used in several existing surveys – as well as PCH-setting (product of time spent in setting and number of people present) and PCH-reach (product of time spent in setting and number of people in close proximity). Heterogeneities in day-dependent distribution of risk across settings are analysed using partitioning and cluster analyses and compared between days and contact measures. Although home is typically the highest-risk setting when PCH measures isolate two-way interactions, its relative importance compared to social and work settings may reduce when adopting a more inclusive contact measure that considers the number and duration of potential exposure events.

**Conclusions:**

Heterogeneities in location-dependent contact behaviour as measured by contact diary studies depend on the adopted contact definition. We find that contact measures isolating face-face conversational or touch interactions suggest that contact in the home dominates, whereas more inclusive contact measures indicate that home and work settings may be of higher importance. In the absence of definitive knowledge of the contact required to facilitate transmission of various respiratory pathogens, it is important for surveys to consider alternative contact measures.

## Introduction

Understanding heterogeneities in population mixing is important for gaining insight into the transmission dynamics of respiratory pathogens and the likely benefit of targeted intervention strategies designed to exploit inhomogeneities in population transmission risk. Agent-based models allow exploration of the effect of detailed heterogeneities in population mixing behaviour on disease spread. Existing agent-based simulations have been used to demonstrate that early epidemic spread is governed by the behaviour of the most social agents (with the largest numbers of contacts) [1]–[3], thus suggestive that identifying and understanding the behaviour of these ‘super-spreaders’ may be crucial for disease containment. Longer term epidemic behaviour, however, is likely governed by the full details of the social mixing structure [1]. It is thus of interest to explore heterogeneity in population mixing, and its interaction with respiratory pathogen transmission risk, empirically. Setting-dependent differences in social mixing behaviour are an important source of heterogeneity in population contact patterns. Contact studies have attempted to quantify variations in the nature of social contacts between social settings [4]–[6], noting that close (and often repeated) contacts are predominantly accrued in home, work and school settings. In addition various intuitive age-dependent effects regarding the role of school and workplace settings, as well as dependence of number of contacts on the type of day (i.e. holiday, weekday or weekend), have been noted [6]–[9]. There exists considerable variation in the results in different contexts [5], [10], [11], indicating that further study is required to characterise the potential range, and consequence, of mixing behaviour.

Traditionally social contact studies measure face-face conversational or touch encounters [4]–[6], [10] and often classify interactions as either touch/non-touch. Indeed assuming that transmission risk is proportional to the age-specific number of contacts has been shown to be consistent with influenza, measles, varicella-zoster-virus and parvovirus [12]–[15] serology in some contexts. However explorations of the relationship between contact intensity and pathogen transmission potential are still limited [16], [17], and there are indications that depending on the pathogen of interest there may be a number factors at play. For example influenza transmission risk within schools has been shown to be heightened compared to the population average [18], but it is unclear whether the enhanced transmission in this setting is solely due to larger per person numbers of two-way interactions or a consequence of larger numbers of persons present in the setting. Attributes of the physical environment such as humidity and ventilation may also play a significant role in determining transmission risk [19]. Exploring differences in the relative influence of different settings for different contact measures may be crucial for understanding both the transmission and epidemiology of non-sexually transmitted close contact infections.

Techniques for capturing reliable mixing behaviour from surveys of contact behaviour are still evolving. Existing contact diary studies are typically ego-centric – recording the behaviour of the survey subject but not their contacts – and thus likely underestimate the true number of contacts. Recent contact diary studies by Smieszek et al. (2011) have highlighted limitations in ego-centric surveys by surveying interactions for both egos and alters in an office-based setting [20]. Different recording tools may also impact on diary accuracy; McCaw et al. (2010) have demonstrated differences in reliability of paper and electronic (PDA) diary tools [21] and Beutels et al. (2006) has explored the utility of web-based recall contact diaries [22]. Designing contact diaries which are easy to use and yield accurate contact data for quantitative studies of social mixing remains a challenge, and the accuracy of different tools in different settings is one aspect that has yet to be explored.

Here we present a second paper on a contact diary study of 65 individuals over three different days of the week using 3 different recording methods, with contact encounters classified as occurring in one of 6 broad setting categories. In addition to considering two-way contact events, we explore other measures of contact intensity and duration – which are more inclusive – that may be relevant for modelling respiratory, or other non-sexual close contact transmitted, disease. We use these data to explore three main questions; the relative accuracy of each diary tool in each setting, variations in setting importance with day of the week, and variations in setting importance with contact definition. Our analysis extends our previous work [6] which focused on the *number* of close encounters with individuals. Our results also complement the recent analysis by Kretzschmar & Mikolajczyk (2009) [5] by exploring the effect of contact definitions on the clustering of the distribution of total contact between setting categories. Our findings have the potential to inform the design of future larger-scale contact studies and agent-based models for epidemic and endemic respiratory disease epidemiology.

## Methods

### Data collection and methodology

Our survey methods are explained in detail in McCaw et al. (2010) [6], and we recall only the main features here. Conduct for this study was approved by the University of Melbourne's Health Sciences Human Ethics Sub-Committee (ID 0721768.2). Written informed consent was obtained from all subjects prior to participation.

We surveyed the contact behaviour of a convenience sample of 65 adults ranging in age from 20 to over 65 years of age, many of whom were associated with the research group responsible for implementing the study. Each participant recorded anticipated contact events for three specified days in a pre-entry questionnaire (Questionnaire). Encounters were recorded prospectively for 2 sets of 3 days (Wednesday, Friday, Sunday) using a cross-over methodology in which participants were randomised to record actual encounters using first a PDA diary (PDA) or paper diary (Paper). We thus have data for 3 diary tools for each of the 3 survey days.

Our contact diaries, which are available in Supporting Information S1, were designed such that participants recorded contacts on a new page for each location visited, allowing us to categorise contact encounters into one of six setting categories; home, other, retail, social, travel and work, as in Edmunds et al. (1997) [4]. Locations listed by the participants were classified according to the intended purpose of their presence. For example subjects employed in a retail environment would be classified as being in a “work” and not “retail” setting during work hours. Similarly subjects visiting the house of a friend would be considered to be in a “social” and not “home” setting. There were no accounts of subjects working from home in during the study.

In addition to recording details of face-face encounters, participants were asked to estimate and record the number of people within arms reach, and in the entire setting, during the period spent in each location visited (see Supporting Information S1 for sample diaries). From this information we extract several different measures of the person contact hours (PCH) accumulated in each setting category; PCH-interaction (cumulative time spent in face-face conversational or touch interaction with contacts), PCH-reach (product of time spent in setting and number of people within arms' reach) and PCH-setting (product of time spent in setting and number of people in setting). Note that unlike some analyses of the numbers of close contacts, contact definitions based on cumulative person contact hours treat repeated encounters with contacts on equal footing with first encounters.

### Statistical analysis techniques

Statistical analyses are performed using *STATA* version *11.2*. We assess potential biases in PCH measures between paper and PDA diaries using a Bland-Altman (BA) test. We report BA differences which quantify the agreement between PCH measures with each diary tool, with (significantly) non-zero BA differences signalling a bias between measures. Kruskal-Wallis rank tests are used as an omnibus test for differences in distributions of PCH-measures (and derivative quantities) across locations. Wilcoxon rank sum tests, with the appropriate Bonferroni correction for multiple comparisons, are used to test for pair-wise significance between ranks. Univariate negative binomial regression analyses are used to explore factors predictive of the number of locations visited.

We treat each person day independently, yielding (a maximum of) 195 subject-day observations for each diary tool. In order to examine heterogeneity in subject mixing across settings for each PCH measure we first normalise the setting specific PCH measures for each subject by dividing the total PCH accumulated in each setting in a survey day by the subject's total daily PCH measure for the same survey day. This normalisation allows us to compare the distribution of PCH between locations (which we refer to as the “contact location profile”) for subject-days with differing daily total PCH, as well as for PCH measures with differing characteristic magnitudes. We explore the heterogeneity in the contact location profile in two ways. We partition the normalised PCH values across settings according to total daily PCH and examine the distribution of PCH between settings in each group. We also cluster data according to contact location profile using average linkage hierarchical clustering routines. The latter approach is similar to that adopted by Kretzschmar & Mikolajczyk (2009) [5]. Note that in contrast to Kretzschmar & Mikolajczyk (2009) our sample size is small and we do not require a two-step approach to cluster our data hierarchically. By clustering based on the *normalised* PCH measures we avoid identifying outlying observations as individual clusters. The optimal number of clusters is assessed with reference to the Cali
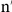
kski-Harabasz pseudo-F measure [23] combined with interpretability of cluster profiles.

## Results

### Comparison of diary tools across setting categories

**Figure 1:**
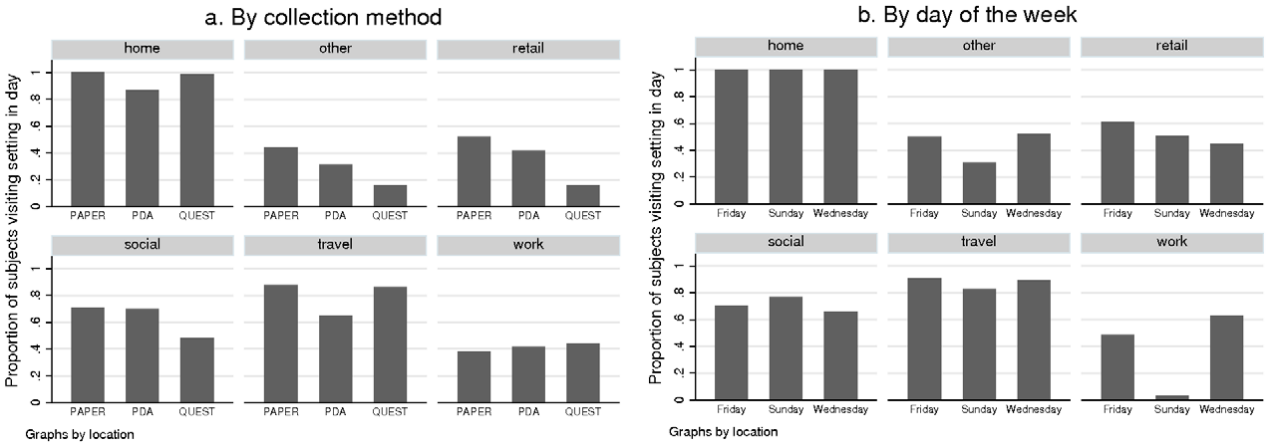
Recorded presence in each setting category. a) Average proportion of subjects reporting presence in each setting category by collection source. b) Proportion of subjects reporting presence in each setting category by day for paper diary only.

Figure 1a) shows the average (over subject-days) proportion of participants present in each setting category as estimated from each diary tool. Whilst no significant difference in recorded presence in the work setting is observed between tools, all other settings reveal a bias between recording tools. The questionnaire is significantly less likely to capture presence in retail (p
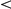
0.0001), social (p
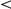
0.0001) and other (p = 0.0012) settings. These trends are consistent with the limitations of the pre-entry questionnaire to capture unplanned absences from work or activities outside the home. We thus narrow further discussion to the two prospective diary tools.

PDA diaries are significantly less likely (p
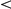
0.0001) to capture presence in travel and home settings. Indeed paper diaries tended to yield higher estimates for the number of setting categories visited daily compared with PDA (BA difference 0.0582 hours, CI(0.380, 0.783)), with differences tending to be larger on weekdays than Sundays. Much of this discrepancy is attributable to recorded presence in the home (BA difference 0.125 hours, CI(0.077,0.173)).

Subjects' total (summed over locations) daily PCH-interaction measures were not significantly different between prospective diary tools, a result which holds for each setting category considered independently. Total PCH-reach measures, however, were larger for paper diaries (BA difference 108.3 hours, CI(65.2,151)) and this difference is significant for all settings except the home, with the largest discrepancy seen in work settings (BA difference 50.185 hours, CI (18.1,82.2)). PCH-setting measured by paper diaries is on average larger in travel (BA difference 39.48 hours, CI(10.9,67.9)) and retail settings (BA difference 69.0 hours, CI(4.19,134)), but no difference between recording tools was found in the other settings.

Ascertainment of subject contact data appears to be superior with paper compared with questionnaires and PDA diaries both for capturing the number of locations visited and the location-specific PCH measures of each type. We thus restrict further analysis to data collected with the paper diaries.

### Frequency of daily presence in settings

The proportion of subject-days for which presence in each setting category is reported is shown in Figure 1(b). The mean number of setting categories visited per day was 3.93 (median 4, IQR(3,5)). This value was significantly (p = 0.0001) lower on Sundays (3.44, median 4, IQR(3,4)) than on Wednesdays and Fridays (mean 4.17, median 4, IQR(4,5)). A univariate negative binomial regression analysis reveals that the number of locations visited is lower on Sundays compared to Fridays (IRR 0.819, CI(0.686,0.978), p = 0.028), however subject characteristics such as age (IRR 0.997, CI(0.991,1.002), p = 0.33), sex (IRR 0.974, CI(0.805,1.178), p = 0.78), household size (IRR 1.015, CI(0.965,1.067), p = 0.56), number of children in the household (IRR 1.024, CI(0.962, 1.089), p = 0.44) and presence of child in the household (IRR 1.045, CI(0.907,1.205), p = 0.53) were not significantly predictive.

Across all days surveyed, participants visited less than 3 setting categories during 10.8% of days, 3–4 setting categories for 56.1% of days and 5 or more settings for the remaining 32.9%. All subjects recorded being present in the home on each day (see Figure 1a & b). Subjects who visited just one other location were never at work, with travel (28%) and other (19%) the most likely alternate setting visited. Participants who visited 5 or more setting categories within a day were more likely to have been in a social setting (96.8%) compared to days in which participants were present in 3 or 4 setting categories (68.8%).

McCaw et al. (2010) found that the recorded numbers of contacts were similar amongst weekdays [6]. We do not find any significant differences between the proportion visiting each setting on Wednesdays versus Fridays (see Figure 1b). Comparison of PCH measures across all three days reveal that Wednesdays and Fridays are similar, except for a significantly larger PCH-interaction measure at work on Wednesday compared to Friday (p = 0.027). Wednesdays and Sundays show the most between-day variation in mixing behaviour. For ease of presentation we therefore limit some of the further discussion of day-dependent contact behaviour to Wednesdays and Sundays.

### Total daily person contact hours

If participants were able to accurately estimate the time-weighted *average* number of contacts within arms reach and within each setting, we would expect that PCH-reach measures would be nested by PCH-setting measures, and similarly (providing conversations occur with contacts in arms' reach) PCH-interaction measures would be nested by PCH-reach measures. Indeed total daily PCH-setting per subject per day was generally larger (median 124 hours, IQR(61.2,248)) than total daily PCH-reach (median 46.4 hours, IQR(27,79.2)) and total daily PCH-interaction (median 10.75 hours, IQR(5.37,20.2)). Although the expected equalities hold true for the majority of observations, there are a number of exceptions (of the range 5–10 per cent) for all settings, highlighting some limitations in subject ability to estimate surrounding contacts self-consistently.

Ratios of PCH measures (for a particular subject and setting) reveal information about the average spatial distribution of contacts within a setting category. The ratio of PCH-interaction to PCH-setting is largest in home and travel settings (Kruskal-Wallis), with home (median 0.51, IQR(0.33,1)) significantly higher than all other settings (p
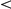
0.0001) except travel (median 0.35, IQR(0.03,1)). High values of PCH-interaction compared to PCH-setting in the home are reflective of the small household sizes of participants (median 3, IQR(2,4)), typical of the Australian population. The high ratio of PCH-interaction to PCH-setting in travel settings is somewhat more surprising and suggests that a large proportion of participants spent travel-setting time in a car/bike rather than on public transportation. Due to our small sample size we cannot comment on whether this trend is representative of the population average.

### Day-day differences in PCH across settings

**Figure 2:**
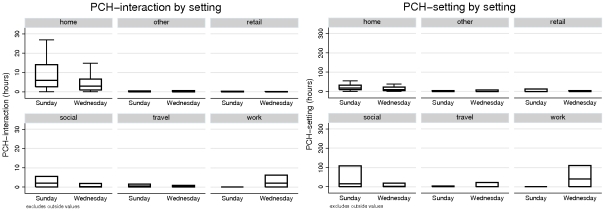
PCH measures for each setting category. PCH-interaction (left) and PCH-setting (right) measures by day and location, in units of hours. Note that PCH-reach distributions are statistically similar to PCH-interaction distributions and omitted here. Also note that outside values are not shown.

We do not observe significant differences between *total* PCH measures on Wednesdays and Sundays. However the distribution of total PCH measure between setting categories shows significant variations depending on day of the week (see Figure 2). PCH-reach and PCH-interaction distributions are statistically similar in each setting, and we therefore focus on comparison of PCH-interaction to PCH-setting measures.

On Wednesdays, PCH-interaction for home ranked highest (median 3 hours, IQR(1,6.67)) but not significantly (p = 0.35) compared to work interactions (median 2.06 hours, IQR(0,6.23)). On Sundays, PCH-interaction is greatest (p = 0.0006) at home (median 6 hours, IQR(2.58,14)), but with social settings (median 2.1 hours, IQR(0.08,5.48)) of subsequent importance (p = 0.013). In contrast, whilst PCH-setting measures in the home rank highest on both days (Wednesday median 10.25 hours, IQR(3.67,22), Sunday median 17.5 hours, IQR(10,33.17)), this ranking was not uniformly significant. PCH-setting measures accrued in the home were not significantly larger than PCH-setting measures accrued in work settings (median 40 hours, IQR(0,110)) on a Wednesday. Similarly home PCH-setting measures were not significantly larger than PCH-setting accrued in social settings (median 15.83 hours, IQR(0.5,108.83)) on a Sunday. Our results indicate that whilst home is typically the setting of highest relative risk when PCH measures are weighted toward personal interactions, risk in social and work settings can become comparatively important when adopting a more inclusive contact measure. Average PCH accrued in travel, retail and other settings are low for all days and measures (see Figure 2).

### Heterogeneity in distribution of PCH across setting categories

**Figure 3:**
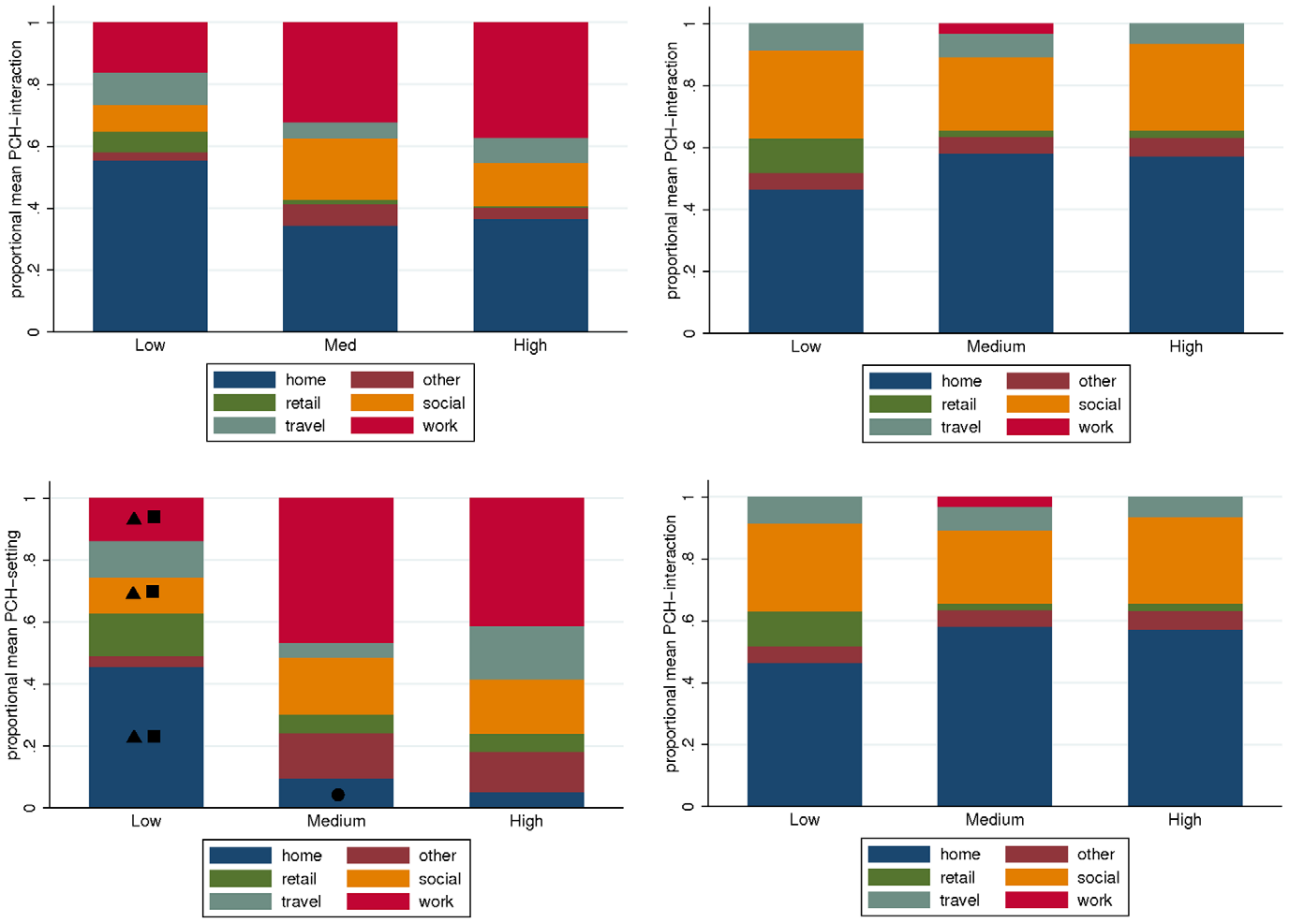
The distribution of total daily PCH between settings. Mean proportion of total daily PCH spent in each setting category for Wednesdays (upper panels) and Sundays (lower panels) partitioned by the total daily PCH. Labels “Low”, “Medium” and “High” indicate daily total PCH in the 0–33 (low), 33–66 (medium) and 66–100 (high) percentile ranges respectively. The left panel shows the relative sizes of the mean PCH-interaction measures and the right panel depicts the relative sizes of the mean PCH-setting measures. The symbols indicate that the trend in relative normalised PCH measure with total-daily-PCH category (i.e. low, medium or high) is signficiant (triangles indicate 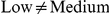, squares indicate 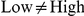, circles indicate 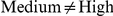).

The distribution of accumulated PCH-interaction hours across locations is not significantly different for people with different total daily PCH-interaction: we find no significant difference in the proportion of total daily PCH time spent in each location for subject-days categorised according to total PCH tertile (see Figure 3). This is not the case for PCH-setting accumulation across settings. In particular, for subject-days with higher total daily PCH-setting, there was a marked decrease in the proportion of overall daily PCH accumulated in the home, counterbalanced by increases in PCH accumulated in social and work settings (see Figure 3). We find that all three PCH measures demonstrate a trend of increasing household size with increasing total daily PCH.

Using PCH-interaction as a proxy for transmission risk would suggest that all hosts, regardless of their total PCH, have similar risk distribution across settings. PCH-setting based risk estimates (and to a lesser extent PCH-reach, results not shown) would identify a relatively lower risk in the home for people with large PCH, offset by increased risk in social and work settings.

**Figure 4:**
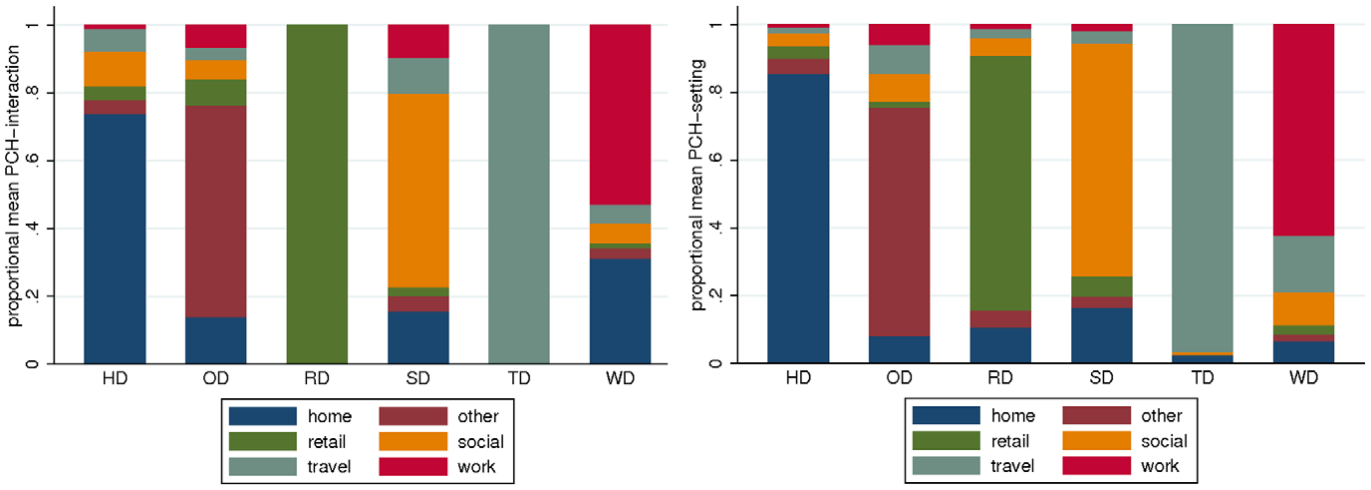
Cluster contact location profiles. Distribution of normalised PCH-interaction (left) and normalised PCH-setting (right) between each of the setting categories for the identified clusters. Clusters are named according to the setting which dominates the total daily PCH; home-dominated (HD), work-dominated (WD), social-dominated (SD), other-dominated (OD), retail-dominated (RD) and travel-dominated (TD).

Both normalised PCH-interaction and PCH-setting distribution between locations are well described by six clusters, with total daily PCH within each cluster dominated by interaction in one of the six setting categories (see Figure 4). We refer to these as home-dominated (HD), work-dominated (WD), social-dominated (SD), other-dominated (OD), travel-dominated (TD) and retail-dominated (RD) clusters. The similarity of cluster contact location profiles amongst clusters identified using PCH-interaction and PCH-setting measures allows us to compare the distribution of subject-days between clusters across contact measures. Given our small sample size and reasonably high dimensional clustering space (over the 6-dimensional setting space) we limit the number of clusters to 6 even though the pseudo-F measure can prefer up to 9 clusters. More refined clustering tends to identify multiple different home- and work-dominated clusters. We note that for both contact measures, the travel-dominated cluster is small, with fewer than 10 subject-days described by this behaviour over the three survey days. The retail-dominated cluster for PCH-interaction is also small. Note that although the retail- and travel-dominated clusters are not well populated, they remain important in hierarchical cluster structures for coarser (and naturally finer) partitioning. Whilst hierarchical cluster analyses often depend sensitively on the similarity and linkage functions adopted [24], we find that our results are relatively consistent for a variety of linkage measures (average, weighted average, centroid and complete, data not shown).

The day-dependent distribution of subjects between clusters largely reflects the results of the analysis of the un-normalised PCH measures. Clustered by normalised PCH-interaction, most hosts are either in the home-dominated or work-dominated clusters on a Wednesday, and home-dominated or social-dominated clusters on a Sunday. Retail-, travel- and other-dominated clusters are small (although the other-dominated cluster is more important on a Friday, containing 8 subjects). Whilst there is no significant difference in total PCH-interaction or subject age between clusters, subject household size is larger for the home-dominated cluster (median 4, IQR(2,4)) than the social-dominated cluster (median 2, IQR(2,4)). This possibly reflects larger amounts of time spent at home for those with families.

We find a more uniform distribution of subjects between clusters based on normalised PCH-setting measures, with all clusters except the travel-dominated cluster containing at least 25 subject days. Far fewer subject-days are assigned to the home-dominated cluster, offset by moderate increases in the number of subject-days assigned to the work-dominated and social-dominated clusters and large increases in the number of subject-days assigned to the retail-dominated and other-dominated clusters (see Table 1). The number of unique clusters that subjects are assigned to over the three survey days is larger for PCH-setting clustering – with individuals classified in a mean of 2.49 (median 3, IQR(2,3)) PCH-setting clusters – than PCH-interaction mixing behaviour (for which the average number of cluster classifications was 1.95 (median 2, IQR(1,2)). Whilst there is no significant difference in household size between PCH-setting clusters, we find that total daily PCH-setting is highest in the work-dominated cluster (median 198, IQR(124,343)). Total daily PCH-setting is also significantly larger in the social-dominated (median 116, IQR(64.8)) cluster than the home-dominated (median 40.9, IQR(29.8, 50.5)) cluster, consistent with social settings generally containing more hosts than home settings.

**Table 1:** Daily and total subject-days in each setting-dominated cluster for clustering on PCH-interaction (left) and PCH-setting (right).

	PCH-interaction clusters						PCH-setting clusters					
Day	HD	OD	RD	SD	TD	WD	HD	OD	RD	SD	TD	WD
Wednesday	22	1	1	12	1	28	7	9	5	10	1	33
Friday	26	7	1	19	0	11	10	10	11	12	0	21
Sunday	43	2	1	18	0	1	15	6	11	31	1	1
Total	91	10	3	49	1	40	32	25	27	53	2	55

## Discussion

We find that paper-based diaries exhibit superior performance to PDA and pre-entry questionnaires in all settings. Interestingly, two-way interactions are more consistently captured with paper compared to PDA diary tools. The reasons for lower reliance in capturing non-conversational/non-touch based interactions may stem from the larger delay reported when recording contact with PDAs [6] combined with a reduced tendency to recall presence of contacts, especially when subjects did not have an emotional or intellectual interaction with the contact [20]. The particularly large discrepancy in PCH-reach measures at work is possibly related to the inconvenience of using this tool [6].

Our results suggest that much of the population is highly mobile, visiting 3 or more setting categories per day, with subjects likely to visit more setting categories on a weekday rather than a Sunday. Most subjects therefore have multiple daily potential sources of exposure to disease. For all contact measures considered inter-day variability is greatest between Wednesdays and Sundays, with contact measures for Fridays often similar to Wednesdays. The significant variations in relative PCH-interaction in home, work and social settings reflect expected trends due to population lifestyle, as well as previously noted day-of-the-week effects [4], [9], providing some validation of our contact diary methodology. We find qualitative and quantitative differences in the distribution of PCH-setting across locations and between days compared to PCH-interaction; home is no longer the setting of dominant contact and work and social settings play a greater role in overall PCH on weekdays and Sundays respectively.

Analyses of agent-based models to assess intervention strategies often conclude that the best strategies target the ‘super-spreaders’ rather than users of any particular location [1]–[3]. We find that the distribution of time spent in two-way interactions between setting categories is relatively independent of the total daily PCH, whereas people with larger total daily PCH-setting tend to accrue more PCH in social and work contexts. These findings suggest that bipartite agent-based simulations of disease spread which associate transmission risk with the total number of people present in a setting may well predict different optimal intervention strategies to models which assume the relevant mode of contact is face-face interactions only.

We find that for each PCH measure, the distribution of PCH accumulated in each setting is well described by six clusters, with each cluster's contact location profile corresponding to PCH dominated by interactions in one of the six setting categories. Kretzschmar & Mikolajczyk (2009)h ave presented a cluster analysis of the distribution of the *number* of contacts across a similar set of setting categories. Although there are some differences between definitions of setting categories, with Kretzschmar & Mikolajczyk (2009) also including a school category and not identifying retail settings as an independent category, Kretzschmar & Mikolajczyk (2009) similarly identify clusters in which contacts are dominated by interaction in home, work/professional and social/leisure and other settings. In addition a cluster with contacts dominated by those made in school settings, a cluster with contacts made in a mixed range of settings, and a cluster in which subjects have low overall numbers of contacts were identified. Whilst we cannot directly compare our results to an analysis of the number of contacts over slightly different setting categories, we find that clustering by PCH-interaction results in similar rankings in the size clusters characterised by work-, home- and social-dominated contact profiles on weekdays and weekends to that noted in Kretzschmar & Mikolajczyk (2009). Our small sample sizes limits us from exploring age-dependent effects in contact location profiles as identified by Kretzschmar & Mikolajczyk (2009). The results presented here provide the additional insight that a broader range of setting categories, including retail and “other”, may be the main source of transmission risk when more inclusive contact definitions are relevant.

Existing comparisons of contact patterns for the contact measures we discuss are limited. A previous analysis of mixing patterns which explored contact rates for different contact definitions including “non-close” contacts [11] concludes that relative mixing rates between age classes are similar for all contact definitions considered. Our findings suggest that relative contact rates for different contact measures may be substantially different if contact encounters are stratified by setting. This complex relationship between setting and transmission risk is likely further compounded by the influence of the physical environment on virus survival [19].

Melegaro et al. (2011) compare paired contact and epidemiological data in order to infer the likely relevant modes of transmission for -zoster-virus. Their analysis suggests that physical contact alone explains the serological data. However the relevance of different contact measures will naturally depend on the pathogen and physical contact is not necessary for other pathogens of interest such as influenza [17]. In addition to further studies simultaneously collecting data on different modes of contact and disease spread, further experimental data on droplet survival and dissemination will help to inform contact definitions appropriate for various viruses under different physical conditions. Better characterisation of the disease transmission risk associated with social contact events will naturally be crucial for developing effective intervention strategies.

We note that our sample probably contains a biased cross-section of occupations, due to the convenience sampling of friends and family of the research group conducting the survey. A number of our subjects are health care workers, who have been shown to have higher than average contact activity [25]. However subjects nominated a range of occupations, including identifying themselves as retired, and we cannot conclude that our study is strongly biased due to occupational activities. We were, however, limited to surveying adults. Children, particularly of school age, would have significantly different mixing characteristics [18]. Our current samples size is too small to make a detailed comparison between mixing patterns in Australia and larger-scale studies in Europe [9], [26] and Asia [11]. We are currently undertaking a similar contact diary study in a larger population which aims to disentangle the effect of environment, including socio-economic status, on contact patterns.

We have shown that characterisation of the relative importance of settings in transmission-relevant social networks, in particular regarding the relative role of home and workplace settings in overall transmission risk, can depend on the contact measure adopted. Our results provide constraints for the aggregate characteristics of bipartite networks capturing location-dependent contact events for a number of contact measures potentially relevant for the transmission of respiratory diseases. Further characterisation of bipartite social networks through larger scale contact studies will be important for understanding the relationship between social structure and disease epidemiology, and will aid our understanding of the variation of disease experience between communities and the role of changing demographics in influencing disease trends.

## Supporting Information

Supporting Information S1 Sample diary cards and instructions as supplied to participants.
(PDF)
