# Supplementary material for: Influence of Contact Definitions in Assessment of the Relative Importance of Social Settings in Disease Transmission Risk
Source: PLoS One. 2012 Feb 16;7(2):e30893. doi: 10.1371/journal.pone.0030893 (PMC3281034; doi:10.1371/journal.pone.0030893)
Supplement: Supporting Information S1 — Sample diary cards and instructions as supplied to participants. (PDF) [file pone.0030893.s001.pdf]

Supporting Information S1

Diary tools

Pre-entry questionnaire and paper diary

Each page of the paper diary corresponded to a single location, in both place and time. That is, if a participant began and ended their study day at home, that location would be represented twice in the participants diary, once in the morning and once in the evening.

A copy of the diary page, and the instructions provided to participants are shown below.

SAMPLE DIARY CARD MONDAY **SUBJECT ID ☐☐☐**

TIME	AM/PM	LOCATION (DESCRIBE)	
APPROXIMATE NUMBER OF PEOPLE IN THIS LOCATION WITH YOU			
APPROXIMATE NUMBER OF PEOPLE AT ARMS' LENGTH AT THIS LOCATION			
IF TIME/LOCATION DETAILS ARE AS PREVIOUS PAGE PLEASE TICK HERE			☐

First Name & Initial (if known)	Occupation (if known)	Home Suburb (if known)	Age OR Range	M/F	Talk or Contact	Length of Contact (approx)	
						Hrs	Mins

University of Melbourne HREC Number: 0721768.1 Version 2, 7TH December 2007

DIARY CARD INSTRUCTIONS

Days of the week:
You are being asked to complete this diary on three separate days. Pages are labelled at the top with the day of the week, and colour coded to help you distinguish them. We define the day as starting when you wake up in the morning, and finishing when you go to sleep.

Locations:
Each time you change your location on a given day to a place where other people are present we would like you to start a new diary page, recording the time of arrival and a description of that location at the top eg home, train, lift, office, tea room, supermarket, movie theatre etc. If you don't have enough room on one page to record all your contacts, continue on to the next page. You can indicate that the location hasn't changed by ticking the box.

Number of People:
Every time you change location, we'd like you to make a rough estimate of the number of people sharing that space with you. This number might range from 0 during a solo car trip to 100 or more in a crowded movie theatre. We would also like you to indicate the approximate number of people who came within arms' length at this location. For example, at the movies, this might refer to the people sitting in the seats immediately in front of and behind you, and those on either side.

Description of Contacts:
Within each new location, we'd like you to identify the people you:

- Talk to (T) – in a two-way or small group exchange involving at least three words
- Contact (C) – any form of skin-to-skin contact, like a handshake or hug

We're asking you to provide us with some minimum information about these people, mainly to be able to identify repeated contacts. Where known, it would be helpful if you could include a first name and initial, occupation and suburb of residence. Regardless of whether you know the person's name, please fill in their gender and approximate age (or estimated range) and the length of time you spent with them. While some people may spend only a few minutes with you, others might occupy the same space for hours, and chat from time to time – in this case, just enter them in the diary once and indicate how long in total you spent together.

Please Note:
Contacts made exclusively by telephone or mobile phone should NOT be recorded.

Thank you once again for taking the time to participate in this study.
If you have any further questions, please contact:

[Research Assistant Name] on [Mobile number]
OR
Dr Jodie McVernon on [Mobile number]

Electronic diary (PDA)

The electronic diary tool used in this study was designed using the VisualCE environment for Windows CE 6 devices. All participants were supplied with a HP iPAQ 112 touch screen device, controlled using the supplied stylus and provided with extensive one-on-one training in how to use the device.

Three screens (‘Location’, ‘Contacts’ and ‘Names’) were used for data entry. For each screen, the user was instructed to complete details from ‘top-to-bottom’. Colour coding was used to guide the user: green for areas for text or data entry, grey for clickable buttons, and red for key information for the user to ensure was correct before entering any information.

The instructions distributed to participants are shown on the next four pages.

The non-linear design of the software minimised the amount of repetition in entering required information. Demographic information for any individual contact was only entered once for the whole three day study period. In fact, upon initiation our research assistant worked with the participant to pre-fill the ‘Names’ database with as many likely contacts for the participant as possible, thereby minimising participants use of the ‘Names’ screen to only those situations in which the contact was unknown or unexpected. Note that in all analyses, only names that were recorded in the ‘Contacts’ database were used.

Participants could enter missed locations (and contacts therein) or missed contacts in an already recorded location by selecting entries from the appropriate table (on the ‘Locations’ or ‘Contact’ screen) and entering/updating fields. For each entry in the database, we recorded time-of-creation and time-of-last-modification. Comparison with user specified time-stamps allowed for analysis of time-delays to entry and the frequency of retrospective entry.

General instructions for using the PDA

1. To turn on the PDA, use the power button on the top right side of the unit.

2. Charge your PDA every night using the supplied power cord, even on days when you are not using it.

3. Never remove the battery – all information on the PDA will be lost instantly.

4. Only use the provided stylus to tap the screen.

5. Keep the PDA in its protective case when you are not using it for entering information.

6. Be patient when tapping buttons – if the screen doesn't change immediately give it a few seconds before tapping again.

7. Use the keyboard button in the bottom right corner of the screen to pull up the keyboard. If you can't read part of the screen, try hiding the keyboard and opening it again.

8. If none of the diary screens are displayed, tap the coloured "Windows logo" in the top left corner of the screen and select "LocationsV2" to start it again.

9. Call [Research Assistant Name] if you have any problems [Mobile number] during business hours.

Locations screen

Work from top to bottom.

Red boxes provide important status information.

Green boxes are for entering information.

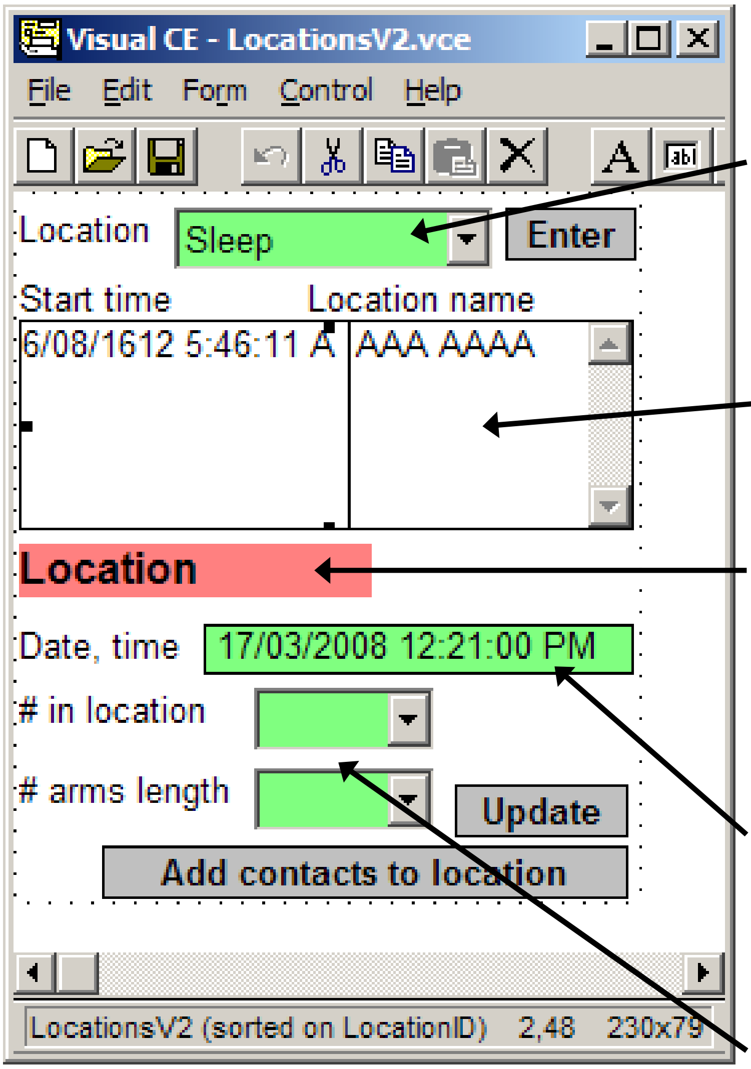

Type your location name here and tap “Enter”. The drop down box lets you use a previously stored name again.

The table allows you to update details from a previous location. Tap once on the relevant entry to update details.

The red box tells you what location you are updating – always check it before entering information in the green boxes below.

Tap on the “Date, time” green box if you need to change the time for this location.

Enter the number of people and the number of people within arms length.

Tap “Update” to see your changes in the table above.

Tap “Add contacts to location” to change to the Contacts screen.

Contacts screen

Work from top to bottom.

Red boxes provide important status information.

Green boxes are for entering information.

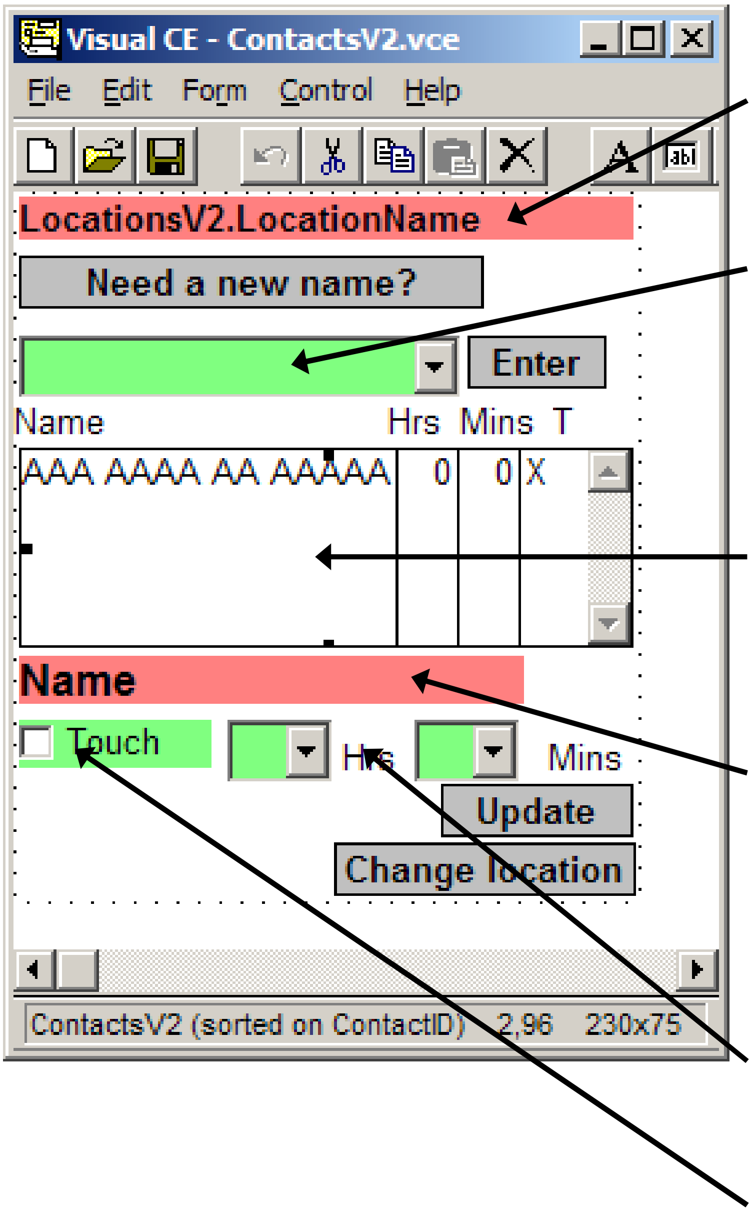

The red box reminds you of your current location.

The green drop down box allows you to select a person to enter as a contact. Select their name and the tap “Enter”. If the person is not in the list, tap the “Need a new name?” button to jump to the Names screen.

The table allows you to update details for another contact in this location. Tap once on the relevant entry to update details.

The red box tells you which contact you are updating – always check it before entering information in the green boxes below.

Enter the total time you have spent with this person in this location.

Select the “Touch” box if you made physical contact with the person.

Tap “Update” to see your changes in the table above.

Tap “Change location” to go back to the Locations screen.

Names screen

Work from top to bottom.

Red boxes provide important status information.

Green boxes are for entering information.

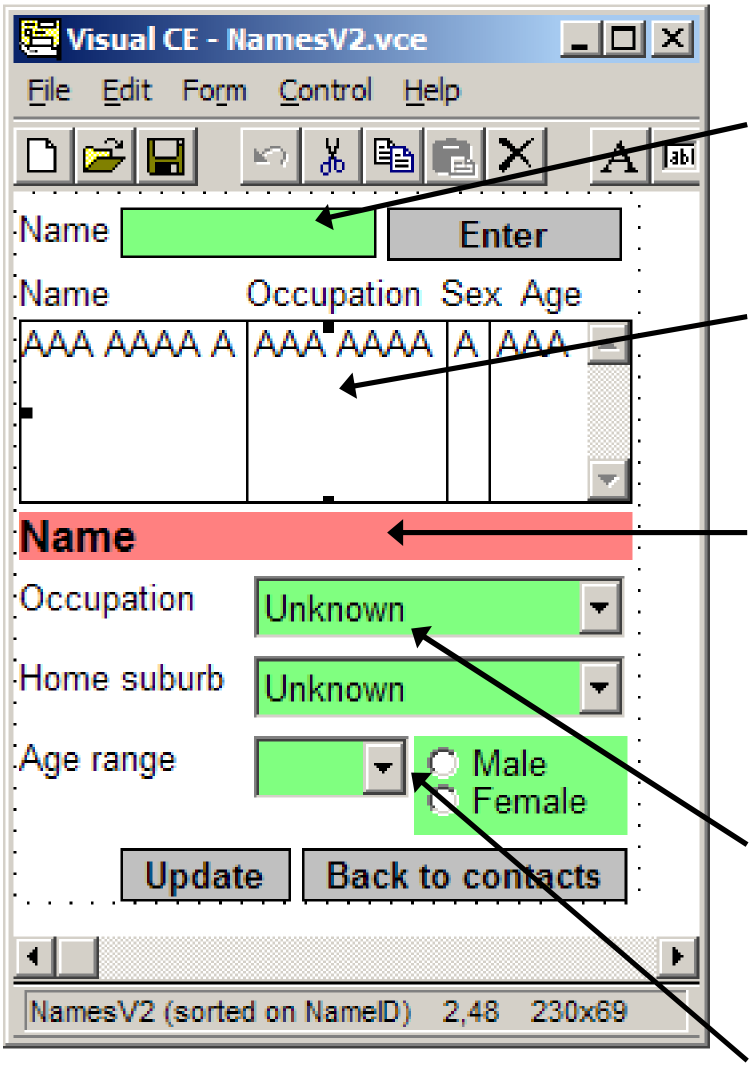

Enter the name of the new person in the green box and tap “Enter”.

The table allows you to update details for a previously entered person. Tap once on the relevant entry to update details.

The red box tells you which person you are updating – always check it before entering information in the green boxes below.

Enter the person’s occupation and home suburb if known. Otherwise leave it as “Unknown”. Previously stored entries are available by using the drop down box.

Select an age range and gender for the person.

Tap “Update” to see your changes in the table above. If you wish to add another person, return to the top of this screen and enter a new name and tap “Enter”.

Tap “Back to contacts” to go back to the Contacts screen where any newly entered people will now be available.
